# Prevalence of positive COVID-19 among asymptomatic health care workers who care patients infected with the novel coronavirus: A retrospective study

**DOI:** 10.1016/j.amsu.2020.06.038

**Published:** 2020-07-11

**Authors:** Nabil A. Al-zoubi, Basil R. Obeidat, Mohammad A. Al-Ghazo, Wail A. Hayajneh, Abdelkarim H. Alomari, Tagleb S. Mazahreh, Ibrahim G. Al-Faouri, Khaled Obeidat, Ali Banni Issa, Abdelwahab Aleshawi

**Affiliations:** a Department of Surgery and Urology, Jordan University of Science and Technology, Irbid, Jordan; b Department of Obstetrics and Gynecology, Jordan University of Science and Technology, Irbid, Jordan; c Department of Pediatric and Neonatology, Jordan University of Science and Technology, Irbid, Jordan; d Department of Community and Mental Health Nursing, Jordan University of Science and Technology, Irbid, Jordan; e Infection Control Unit, King Abdullah University Hospital, Irbid, Jordan

**Keywords:** Coronavirus, Health care workers, COVID-19, KAUH

## Abstract

**Background:**

Limited information is available about COVID-19 infections among health care workers. Sensitive detection of COVID-19 cases in health care workers is crucial for hospital infection prevention policy, particularly for those who work with vulnerable patients. The aim of this study is to describe the prevalence of positive COVID-19 among asymptomatic health care workers who took care of patients with COVID-19 during the pandemic.

**Methods:**

This retrospective study included all health care workers at King Abdullah University Hospital who take care of patients infected with COVID-19 patients from March 18, 2020 to April 29, 2020. They were tested for COVID-19 infection by use of real-time reverse-transcriptase rRT-PCR on samples from nasopharyngeal swabs.

**Results:**

A total number of 370 health care workers were screened. The majority were nurses followed by physicians and other personnel. This study showed that all asymptomatic health care workers were tested negative for COVID-19Q.

**Conclusion:**

Unexpectedly, the prevalence of positive COVID-19 among asymptomatic health care workers who take care of patients infected with the novel coronavirus was 0%. This result must be cautiously interpreted. Further studies are needed in order to find effective strategy of screening health care workers to insure a safe working environment.

## Highlights

• The prevalence of positive COVID-19 among asymptomatic HCWs was 0%.
• The result should not give us a false sense of security.
• Improvement of surveillance and protection of HCWs is important.

## 1 Background

COVID-19 is a serious illness that currently has no known treatment or vaccine and is spreading in an immune naive population. Deaths are rising steeply, and health systems are under strain [1]. Since the first reported case of COVID-19 in Wuhan, China, at the end of 2019, COVID-19 has rapidly spread throughout China and has also involved many other countries despite global effort s to prevent its spread [2]. On March 2, 2020, the first case of COVID-19 was diagnosed in Jordan to a patient who came from Italy. Total number of cases reached 448 by May 1, 2020.113 of them were admitted to King Abdullah University Hospital (KAUH) which is the only hospital that deals with COVID-19 patients in the north of Jordan.

Health care workers are essential workers defined as paid and unpaid persons serving in health care settings who have the potential for direct or indirect exposure to patients or infectious materials [3]. It is critical to ensure the health and safety of HCWs, both at work and in the community [3]. They are at increased risk of contracting communicable diseases, including droplet-spread respiratory viruses, because of their high level of exposure at work [4]. The idea of implementing a screening regimen to determine the prevalence of COVID-19 among our HCWs using real-time reverse-transcriptase RT-PCR on nasopharyngeal samples was to assess the subclinical transmission of the disease among HCWs, evaluate our protective measures and to help in making decisions regarding staffing and protection of HCWs in the hospital during the COVID-19 pandemic. To the best of our knowledge, very few studies have been conducted about the prevalence of COVID-19 among asymptomatic HCWs during coronavirus pandemic.

## 2 Methods

This study was approved by IRB of Jordan University of Science and Technology. It was a retrospective single center study. Inclusion criterion were all asymptomatic HCWs including physicians, nurses and other personnel (housekeepers, porters and administrative staff) who were assigned to deal with COVID-19 patients at KAUH between March 18, 2020 to April 29, 2020. The doctor/patient ratio was 1/20 while nurse/patient ratio was 1/5–10. A total number of 370 HCWs were voluntarily screened for COVID-19 by nasopharyngeal swabs between 22 and April 29, 2020. Sample collection was taken by our trained team and the swabs were tested for COVID-19 RNA using real-time reverse-transcriptase rRT-PCR. Data on age, sex and occupational categories were also analyzed.

HCWs who had any symptoms suggestive of COVID-19 have been excluded from this study. HCW with comorbidities (diabetes, hypertension, respiratory diseases and other chronic medial illness) and pregnant ladies were not included because they were already excluded from work during this period. This study was conducted according to STROCSS 2019 guideline [5].

## 3 Results

Total numbers of HCWs were 3000 which were reduced to about 50% (1500) during this pandemic. A total number of 385 HCWs were involved in direct contact with COVID-patients. Only of 370 HCWs were screened while 15 were unable to do the test. The asymptomatic HCWs screened for Covid-19 include the following occupational categories: 61.62% nurses (228 of 370), 31.35% physicians (116 of 370) and 7.02% other personnel including; housekeepers, porters and administrative staff (26 of 370). The average age was 32.02 years with the majority of HCWs was males 67% (248 of 370). This study showed that all asymptomatic HCWs were tested negative (Table 1). Therefore, unexpectedly, the prevalence of positive-COVID-19 among asymptomatic HCWs who take care of patients infected with the novel coronavirus was 0%.

**Table 1:** Demographic data of health care workers.

Category	Nurses	Physicians	Others^a^	Total
Number	228	116	26	370
Percentage	61.62%	31.35%	7.02%	100%
Average Age (years)	32.96	28.12	35	32.02
Gender Male/Femal	149/79	83/33	16/10	248/122
Number of Positive rRT-PCR Results	0	0	0	0

a Housekeepers, Porters, Administrative staff.

## 4 Discussion

Since December 2019, the world has been in the grip of the severe acute respiratory syndromecoronavirus 2 and the disease it causes, coronavirus disease 2019 (COVID-19) [6]. Since then, the spread of COVID-19 has increased exponentially, with the World Health Organization (WHO) declaring a pandemic on 11 March [7].

They are at high risk of morbidity and mortality due to health care-associated infections [8]. It has been estimated that 10% or more of all those infected with COVID-19 in some European countries are HCWs [4]. In Italy, HCWs experienced high rates of infection and death [9].

Little is known about the effectiveness of personal protective equipment (PPE) for HCWs who take care of patients infected with COVID-19 [10]. On the other hand, other studies showed that the prevalence of COVID-19 amongst HCWs will depend upon a range of factors, including the availability of PPE, the healthcare setting and access to testing.

How well the country handles the Covid-19 crisis depends largely on how effectively health workforce is used [11]. But, it is also necessary to protect the health and safety of this essential national workforce [4]. Therefore, our local infection control committee started before the beginning of this pandemic in Jordan to educate HCWs about caring for COVID-19 patients regarding hand hygiene, donning and doffing PPE. Types of PPE used in KAUH were: gloves, N95 masks with tight seal around mouth and nose, face and eye protection including face shields and goggles, clothing which includes gowns, aprons, head covering, and shoe covers. Moreover, we started to establish policies and modify regulations to protect HCWs during these challenging times. As a result, our hospital started to operate at less than half capacity in order to decrease workload and to prioritize resources. In order to minimize the risk to the HCWs, only necessary procedures were allowed. Aerosol generation procedures were limited to negative pressure rooms, they were undertaken by most expert doctors or nurses available and only necessary HCWs were present in the room. HCWs with comorbidities (DM, HTN, respiratory diseases and other chronic medial illness) and pregnant ladies were exempted from work during this period. We also adopted a policy which allows every HCW to take two weeks of home self-isolation after finishing working shift (We divided the 385 HCWs into 14 groups each group contains about 27 personnel who work 24 h. This would provide about 2 weeks of home isolation for each HCW). Moreover, we separated wards that could be contaminated with the virus from other low risk facilities and we minimized the time of contact between HCWs and infected patients by limiting unnecessary procedures, decreasing the communication times between infected patients and HCWs provided that the patient care is not affected and by trying as much as possible to do many tasks in the same round as taking vital signs, giving medications, distributing meals and doing beddings.

Early in the pandemic crisis in Jordan (first 5 weeks) there were shortage of PCR-kit due to supply chain problems, the priority was to test only suspected patients and symptomatic HCWs. Taking into account that not all cases are symptomatic and some people may be infectious before they develop symptoms, many HCWs were worried about their risk of exposing family members to the virus [4]. As a result, there was a need for HCWs screening protocol that provides data base about the prevalence of COVID-19 infection among our staff. Therefore, a screening regimen has been proposed for all HCWs who work in direct contact with COVID-19 patients including physicians, nurses and other personnel.

Given the high prevalence of mild clinical presentations that may go undetected [5] and after the availability of PCR-kit, we decided to changed our strategy to be sure that our HCWs are free of infection by both the PCR test and home-isolation period. So, we started to screen all asymptomatic HCWs with any history of contact with COVID-19 patients.

We think that the advantage of having an objective regimen by lab test will help in decreasing the stress and worries of the HCWs about their risk of having the virus [4], to reduce the risk of transmitting COVID-19 from HCWs to their colleagues or to other non-COVID-19 patients and to be able to formulate policies regarding workflow, especially in the absence of standard best practices. Moreover, this may help health care providers in other institutions to develop policies regarding HCWs during similar pandemics.

As mentioned above, some studies showed that about 10% of all those infected with COVID-19 in some European countries are HCWs [4]. However, in this study, we found that the prevalence of positive-COVID-19 among asymptomatic HCWs who take care of patients infected with the novel coronavirus was 0%. Although this result could be in part due to our policies and protective measures, it was unexpected and against our assumption as HCWs are high risk group. Potential explanations for this difference in the result are; firstly, the majority of admitted patients to our hospital were asymptomatic 88% and detected by epidemiological investigation teams as patients are most infectious when they are symptomatic. Secondly, rRT-PCR test has risk of eliciting false-negative results especially in asymptomatic cases due torelatively lowviral loads. Additionally, we used nasopharyngeal swab sampling. However, the optimum sample types during infections caused by COVID-19 remain to be fully determined and require expertise [12].

The findings of this study have to be seen in light of some limitations. The first is the limited number of COVID-19 patients admitted to our hospital. The second limitation concerns the timing of screening which was 4 weeks after the admission of the first COVID-19 patient, during this period, HCWs may have been infected with the virus at some point and recovered by the time of screening. Additionally, this is an empiric way of screening due to lack of previous research studies that address this subject.

It is worth mentioning that among the 385 HCWs assigned to deal with COVID-19 patients only two nurses were infected. First case was male aged 27 years who was diagnosed after he developed symptoms at the beginning of his fist shift, most probably that he had infected outside the hospital. He was excluded from the study because he was symptomatic and the inclusion criterion was only asymptomatic HCWs. The second case was 28-year-old asymptomatic female, she diagnosed by rRT-PCR due to history of contact with her infected colleague and she was excluded from the study as the source of infection was outside the hospital. This represents only 0.5% of total HCWs.

Importantly, this is a rapidly moving topic of research. Therefore, our result must be cautiously interpreted and should not give us a false sense of security regarding the prevalence of positive COVID-19 among HCWs. Our perspective is to improve surveillance of HCWs and to identify the best approach to protect HCWs in order to assure source control to insure a safe working environment.

## 5 Conclusion

Unexpectedly, the prevalence of positive COVID-19 among asymptomatic HCWs who take care of patients infected with the novel coronavirus was 0%. This result must be cautiously interpreted. Further studies are needed in order to find effective strategy of screening HCWs to insure a safe working environment.

## Ethical approval

Institutional approval was obtained from the Institutional Review Board at Jordan University of Science and Technology.

## Sources of funding

No funding

## Author contribution

All authors contributed significantly and in agreement with the content of the article. All authors were involved in project design, data collection, analysis, statistical analysis, data interpretation and writing the manuscript. All authors presented substantial contributions to the article and participated of correction and final approval of the version to be submitted.

## Registration of research studies

Researchregistry5631.

## Guarantor

Nabil Al-zoubi

## Consent

Written informed consent was waived due to the retrospective nature of the study.

## Provenance and peer review

Not commissioned, externally peer reviewed.

## Declaration of competing interest

The authors declare that they have no competing interests.
